# Innovation in Boundary-Spanning Technology M&A: A Fuzzy-Set Analysis of Diversity Dynamics

**DOI:** 10.3389/fpsyg.2022.766166

**Published:** 2022-07-11

**Authors:** Meng Qi, Xiaoyi Li, Wanqiu Wang

**Affiliations:** College of Economics and Management, Beijing University of Technology, Beijing, China

**Keywords:** boundary-spanning technology M&A, top management teams, social categorization perspectives, information/decision-making perspectives, fsQCA

## Abstract

The growing literature on organizational innovation has drawn attention from net effect and contingent effect of diversity-related factors in the context of top management teams (TMTs) to their complementarity and interaction in the form of configurations. In post-boundary-spanning technology mergers & acquisitions (M&A), the integration between multi-boundary knowledge and resources necessitates effective communication and cooperation within TMTs that display heterogeneous attributes. Therefore, this study integrates two popular theoretical perspectives from the diversity literature (social categorization perspectives and information/decision-making perspectives) in order to explore the configurational patterns of factors stimulating innovation in boundary-spanning technology M&A (BTM&A). In accordance with this theoretical objective, this study adopts fuzzy-set qualitative comparative analysis for the purpose of examining the complex combinations of five antecedent conditions (functional experience diversity, boundary-spanning experience diversity, faultline strength, number of subgroups, and subgroup balance) based on a BTM&A sample of firms in the Chinese A-share market during the period 2007–2018. Findings from this analysis indicates four configurations of diversity-related factors (the dominated multiple diversities; the non-aligned multiple diversities; the balanced similarity; and the aligned single diversity) which lead to superior innovation in BTM&A. This study fills a gap in the literature vis-à-vis the causes of innovation in BTM&A and provides novel insights for management practitioners to take appropriate countermeasures with regard to TMT diversity.

## Introduction

Boundary-spanning technology mergers and acquisitions (BTM&A) are an important way to integrate and exchange multidisciplinary resources and knowledge across organizational knowledge boundaries, and they play an important role in organizational innovation (Colman and Rouzies, 2019). BTM&A provides substantial opportunities for knowledge transfer across different organizations, but it can be difficult to manage and use such diversified knowledge. According to upper echelons theory, the top management team (TMT) plays a key role in managing diversified knowledge and making decisions related to innovation (Chen et al., 2020). Organizations encourage TMTs to include various deep-level attributes (e.g., functional expertise and boundary-spanning experience) to develop divergent thinking and to understand and solve issues in the innovation process (Harvey, 2013). Research into information and decision-making also supports the idea that diversity in deep-level attributes provides a large pool of knowledge and allows for the development of non-redundant peer networks, which can increase access to unique knowledge-facilitating innovation (Buyl et al., 2011).

Although the potential value of deep-level TMT diversity is clear, the empirical literature on the performance benefits of TMT diversity has been decidedly mixed. Studies have found a positive relationship between TMT diversity and innovation in some cases (Van Knippenberg et al., 2011) and a negative or null relationship in other cases (Tuggle et al., 2010). A number of reasons for this inconsistency in the findings have been identified. First, diversity research mainly focuses on unidimensional TMT diversity rather than the alignment of multiple diversity characteristics (Chen and Wang, 2021). Compared with the convergence of multiple attributes, unidimensional diversity has relatively weak predictive power for team innovation (Lau and Murnighan, 1998), because it does not reflect the interaction among members according to different attributes (Bezrukova et al., 2007). Recent studies have therefore paid equal attention to single-characteristic diversity and the convergence of multiple attributes (Qi et al., 2022).

Second, different theoretical perspectives in diversity research are unconnected (Qi et al., 2022). There are two main traditions in diversity research: social categorization perspectives and information/decision-making perspectives (Van Knippenberg et al., 2004). Social categorization perspectives hold that diversities are used as a basis for categorization that produces subgroup bias within teams. Subgroup members in the same subgroups share similar characteristics (such as cognitive styles and cognitive ability), resulting in a high level of identification within the subgroup. In contrast, the differences between out-subgroups tend to be salience, which leads to a sense of hostility and reduces team cohesion, and further inhibits boundary-spanning innovation (Yang et al., 2020). In contrast, information/decision-making perspectives indicate that diversities may lead members to identify and access differentiated information using their own unique cognitive styles. By integrating and processing this differentiated information, the team is able to access a wider range of knowledge, skills, and resources, which in turn has a positive impact on innovation (Chen et al., 2019).

As these two theoretical perspectives have opposite theoretical implications, they are often studied in isolation, whereas the categorization–elaboration model indicates that these two theoretical perspectives conjoin with each other. Specifically, the positive relationship between diversities and the formation of creative ideas can be impeded by intra-group bias caused by social categorization (Van Knippenberg et al., 2004). Qi et al. (2022) indicate that failing to consider the interaction between different theoretical perspectives of diversity may have led to such inconsistent findings (D’Arcy and Devaraj, 2012).

Third, since organizational boundary-spanning innovation activities are complex, they usually involve different kinds of diversity and different theoretical perspectives, and neglecting these may lead to underestimating the complexities involved (Cabrilo and Dahms, 2020). Most diversity studies, however, focus only on the “net effect” or “contingent effect” of one or a few antecedents rather than emphasizing conjunction, equifinality, and asymmetry (Van Knippenberg et al., 2004). Configurational research argues that the effect of diversity on a negative outcome is not merely the inverse of its effect on a positive outcome, and vice versa. It is therefore important to consider the configurational relationship between variables when determining their overall impact because their transmission mechanisms can be functionally equivalent (Bell et al., 2014).

With regard to the issues discussed above, this study explores the configurations of both unidimensional TMT diversity (boundary-spanning diversity and functional diversity) and the alignment of multiple diversity characteristics (faultlines, number of subgroups, and subgroup imbalance) on innovation during BTM&A based on the integration between social categorization and information/decision-making perspectives. The purpose of the research is twofold: (1) To explore the core conditions and peripheral conditions of innovation during BTM&A based on different configurations of diversities; and (2) to explore the integration of social categorization and information/decision-making perspectives based on the inter-connection between different antecedents in the field of diversity.

## Theoretical Analysis

### Unidimensional Top Management Team Diversity

In order to discern the attributes of unidimensional diversity in the background of BTM&A, this study focuses on two types of diversity: boundary-spanning diversity and functional diversity. This is because boundary-spanning innovation relies on the accumulation of professional experience by senior executives. The functional background and boundary-spanning experience of TMT members are important features of their professional experience. More precisely, TMTs with a heterogeneous functional background offer different views on internal functional departments (Kaplan, 2011), which affects internal cooperation and the integration of heterogeneous boundary-spanning knowledge. TMTs with different boundary-spanning experience have different internal and external network resources (Wiklund and Shepherd, 2003), which helps organizations to identify different technical knowledge objectives for the purpose of enhancing boundary-spanning innovation.

In a comprehensive review of the literature, information/decision-making perspectives and social categorization are commonly used to explain the effect of diversity on innovation (Van Knippenberg et al., 2004). Based on information/decision-making perspectives, TMTs can be viewed as the sum of their members’ work experience (Cheung et al., 2010). The knowledge or information benefits that reside in teams with diverse work experience can be used to develop a team or organizational innovation through information processing (Chua, 2013). More precisely, team members with diverse work experience are likely to exploit, combine, build on and experiment with these different ideas from various perspectives (Shin et al., 2012). Consequently, team members have more choices, plans, and products within this cognitively diverse environment (Wang et al., 2016).

In contrast, social categorization perspectives assert that similarities and differences are used to categorize oneself and others into in-groups or out-groups (Van Knippenberg et al., 2004). Perceiving others as belonging to an out-group may give rise to social bias between the in-group and out-group, which can cause negative stereotypes, conflict, or animosity to the surface (Thatcher and Patel, 2012). This further hampers within-team information exchange and collaboration between team members. Similarly, some empirical studies demonstrate that diverse work experience has a negative effect on innovation (Tuggle et al., 2010).

### Faultlines

Faultlines are defined as hypothetical dividing lines which divide a team into subgroups according to the alignment of multiple diversity characteristics (Lau and Murnighan, 1998). when multiple characteristics converge, teams are divided into different subgroups on the basis of perceived high intra-subgroup similarities and inter-subgroup differences (Cooper et al., 2014). This faultline analogy is in line with the comparative fit social categorization (Brewer and Miller, 1984) which refers to the extent to which similarities and differences are perceived within and between subgroups, which is linked to divisions across social categories (Homan et al., 2007).

An example to explain the nature of a faultline is that imagine a four-member TMT where two members have a marketing background and boundary-spanning experience, while the other two have an HR background and no boundary-spanning experience. Such a situation is likely to create strong faultlines, thereby giving rise to two distinct subgroups (Vora and Markoczy, 2012). Now imagine a different four-member TMT with different but overlapping attributes. Such a team is less likely to have clear subgroup boundaries. Both teams have equally diverse experience, but the former has a stronger faultline.

Based on the nature of the comparative fit, faultlines create salient in-subgroup/out-subgroup membership, thereby enhancing information polarization between subgroups (Lau and Murnighan, 1998). The “us versus them” mentality of subgroups formed through strong faultlines not only leads to in-subgroup favoritism and out-subgroup hostility (Lau and Murnighan, 2005) but also TMT fragmentation vis-à-vis boundary-spanning knowledge. More precisely, such a mentality can cause individuals to hold negative stereotypes toward boundary-spanning knowledge from the out-subgroup and display blind devotion to the knowledge generated by their own subgroup. In such situations, subgroups tend to display intolerance toward diverse ideas vis-à-vis dealing with boundary-spanning knowledge proposed by other subgroups (Lau and Murnighan, 1998). Such a barrier to diverse ideas is detrimental to the team or organizational innovation.

Although most faultline studies focus on the potentially negative effect of subgroup comparative fit, this does not always hold in practice. More precisely, bias and conflict can be avoided when subgroup differences are framed in a positive manner (Hornsey and Hogg, 1999). Faultlines are particularly effective at reducing or preventing innovation-related uncertainty or risk in the P-M&A stage, because subgroup categorization depersonalizes the perceptions and behaviors of individuals, displaying the desire to conform to subgroup prototypes that clarify how individuals should behave and interact with each other (Hohman et al., 2017). Such categorization makes behavior predictable and allows individuals to receive support and validation for their knowledge in their own subgroups. With such support, they may openly engage in discussions concerning boundary-spanning knowledge across subgroups (Qu and Liu, 2017), which can enhance individuals’ confidence in such matters. Such a finding is in line with the literature on categorization influence which shows that knowledge exchange in different teams depends on the extent to which team members are offered social support (Bragg and Allen, 1972). Furthermore, diverse experiences across subgroups along with faultlines can encourage mutual subgroup distinctions (Bezrukova et al., 2009). In practice, some team members are more concerned with dissimilar ideas about boundary-spanning knowledge from out-subgroups than from their own subgroup because they desire subgroup distinction. Hence, they prefer to be dissimilar to out-subgroup members rather than in-subgroup members (Cooper et al., 2014). Expressing ideas across subgroups, in turn, promotes team or organizational innovation.

### Subgroup Imbalance

According to social categorization perspectives, team members tend to categorize themselves and others into subgroups based on similarities and differences between one another, with ensuing within-subgroup favoritism and out-subgroup hostility (Qi et al., 2022). When the relative distribution of subgroup members is uneven, leading to the existence of minority subgroups or majority subgroups in teams, this is called *subgroup imbalance*. The opposite situation is called *subgroup balance* (Carton and Cummings, 2012). The minority subgroups or majority subgroups may have heterogeneous expectations of what an ideal member of this superordinate team turns to be.

According to the in-group projection of social categorization perspectives, the subgroup size decides the relative prototypicality of the superordinate team. The larger the subgroup majority, the more likely they will form the prototype (Hogg et al., 2012). The relative prototypicality for the superordinate team leads to overlap between the subgroups and the superordinate team and increases bias between subgroups (Verkuyten and Martinovic, 2016). The more prototypical the subgroup, the more positive the evaluation of its in-subgroup (Wenzel et al., 2007). In the majority of subgroups, members tend to evaluate their own subgroup as normative with regard to the common superordinate team. The out-subgroup (less prototypical) is viewed as deviating from this normative standard and thus considered inferior (Waldzus and Mummendey, 2004). Mullin and Hogg (1998) assert that the process of assimilating oneself to the prototype for the superordinate team validates the self-concept of individuals. As a result, innovation-related uncertainty or risk in the P-M&A stage is reduced because being the prototype enhances the self-subgroup-team fit which can improve perceived entitativity (Reid and Hogg, 2005).

With regard to the effect of subgroup imbalance on team innovation, some studies suggest that teams are more effective when subgroups differ in size (Qu and Liu, 2017), because the larger subgroup, as the prototype, will push information processing forward (Xie et al., 2015) and encourage diverse boundary-spanning knowledge to converge. In support of this view, the information process assumptions proposed by Peterson and Nemeth (1996) indicate that teams can better achieve a common understanding when they include differently sized subgroups, as the larger knowledge-related subgroups offer a dominant frame of reference. In contrast, teams with equally sized subgroups are more likely to openly conflict vis-à-vis managing boundary-spanning knowledge (Carton and Cummings, 2012), which will limit information exchange across subgroups, as the strong competition among subgroups enhances the salience of subgroup boundaries (Carton and Cummings, 2012).

However, some argue that teams are more effective when subgroups are equal in size (Crucke and Knockaert, 2016) because each subgroup is equally represented in the superordinate team. Consequently, the information and ideas of each subgroup will be equally considered. Such a view is in line with Schweiger and Finger (1984), who find that unique information is less likely to be marginalized when it comes from a subgroup that is as well represented as other subgroups in the same team. As a result, having equally sized teams should lead to better decisions, which further enhances team innovation (Crucke and Knockaert, 2016).

### Number of Subgroups

According to social categorization perspectives, team efficacy is dependent on the number of subgroups. Some studies note that having two subgroups is suboptimal as such a configuration yields the strongest in-subgroup and out-subgroup stereotypes (O’Leary and Mortensen, 2009). When two subgroups are nested in the common superordinate team, team members clearly know who is the in-subgroup and who is the out-subgroup (Qu and Liu, 2017) and thus may adopt an “us vs. them” mentality. Such a finding is consistent with Thatcher and Patel (2012), who observe that teams with more than two subgroups experience less divisive outcomes than teams with two subgroups.

According to the information/decision-making perspective, the more subgroups, the broader the use of diverse knowledge (Qu and Liu, 2017). Increasing the number of subgroup-based resources can increase team elaboration of boundary-spanning knowledge because there is less of an “enemy” threat to each subgroup and subgroup members feel less threatened. As a result, diverse information and ideas from different subgroups are more likely to be processed in a constructive manner (Cooper et al., 2014). These benefits, in turn, promote a team or organizational innovation.

However, an opposite view indicates that increasing the number of subgroups makes it harder for a unified mental model to form. More precisely, the number of subgroups increases the variety of external information inputs. Members become less likely to integrate those inputs into a coherent whole and instead devote more time to knowledge from their own subgroup (Chung et al., 2015). Mathieu et al. (2000) state that the convergence of mental models plays an important role in facilitating team innovation because it sets up a coherent frame of reference through which diverse boundary-spanning knowledge can be integrated.

### The Configurational Model

The mixed findings from previous studies on unidimensional TMT diversity, faultlines, number of subgroups, and subgroup imbalance are likely due to the omission of key combined diverse factors resulting from the use of different theoretical lenses. Given the conceptual overlaps and logical connections between multiple social categorizations and information/decision-making perspectives, their configurational model provides a comprehensive understanding of the antecedents of boundary-spanning innovation. Taken together, this study adopts the fuzzy-set qualitative comparative analysis (fsQCA) method to explore the interconnected causes underlying organizational boundary-spanning innovation together with social categorization and information/decision-making perspectives. The model consists of five components (boundary-spanning diversity, functional diversity, faultline strength, number of subgroups, and subgroup balance) that are used to explore antecedent situations for high organizational boundary-spanning innovation.

## Sample and Data

### Sample

BTM&A is defined according to the following criteria:

(i) Announcements of M&A in which the primary goal of M&A is obtaining technology, patents, or technical personnel from the target company are defined as technology M&A;
(ii) Boundary-spanning refers to the scope of business, main products, and core technologies of the two parties differing from one another;
(iii) Technology M&A is a motive for boundary-spanning if the lead company intends to join the target company’s technology field or combine its existing technology with the target company’s technology so as to upgrade existing products or technology, or enter into a new field.

In addition, the following exclusion criteria are applied to BTM&A cases involving listed companies in the Chinese A-share market:

(i) The first announcement of the M&A must have been made between 1 January 2007 (when information disclosures for companies were completed) and 31 December 2018 (to ensure patent data 2 years post-boundary-spanning M&A is available);
(ii) Lead companies without executive information are excluded;
(iii) Failed transactions and uncompleted cases are excluded;
(iv) Merged companies that did not make patent applications prior to the M&A are excluded.

This left 85 listed companies in the sample. Patent data come from the China Patent database, while manager information (including functional background and work experience) comes from the China Stock Market & Accounting Research Database (CSMAR) and corporate annual reports.

### Variable Measurement

#### Boundary-Spanning Technology M&A Innovation Performance

Invention patents are more representative of a company’s high level of technological innovation than utility models and design patents (Tong et al., 2014). At the same time, in order to alleviate the issue of lagging innovation, BTM&A innovation is measured using the number of invention-based patent applications in the second year P-M&A.

#### Functional Experience Diversity

Using the CSMAR database, functional experience is classified into nine groups – these are production, design, manufacturing, finance, human resources, procurement, administration, sales, and finance. The heterogeneity of functional background is measured according to the number of professional background experiences. For example, an individual with functional experience involving two departments (e.g., the design department and department of administration) is given a score of 2.

In line with prior studies, Blau’s index (Blau, 1977) (as shown in Eq. 1) is used to calculate the degree of functional experience diversity.


(1) $$B=1-\sum_{i=1}^{n}P\,i^{2}$$

Where *Pi* refers to the percentage of functional experience; *i* is a TMT, and n equals the degree of functional experience. The value of B falls between 0 and 1. The greater B is the more functional experience diversity in the TMT.

#### Boundary-Spanning Experience Diversity

In line with Christopher et al. (2010), the boundary-spanning experience of executives is classified as follows. A score of 1 is given if the executive has work experience in a single company; a score of 2 is given if they have cross-organization experience rather than cross-industry experience, and a score of 3 is given if they have cross-industry experience. We also apply Blau’s index to measure the degree of boundary-spanning experience diversity.

#### Faultline Strength

The strength of faultlines that can potentially split a team into subgroups is calculated using the average silhouette width (ASW). The ASW method uses cluster analysis to identify a range of possible subgroups based on the attributes of team members (Meyer and Glenz, 2013). This cluster analysis starts with a small team configuration, where each team member is placed in own individual subgroup. Members with similar attributes are then aggregated together to form larger subgroups. At each step, the two most similar subgroups are merged. Finally, all subgroups are merged. ASW values are produced at each stage of the clustering process. ASW denotes the average profile width calculated for each team member, which quantifies the fit of each team member in their subgroup. Equation (2) shows the equation used to calculate the ASW.


(2) $$S\,\left(i\right)=\left(b_{i}-a_{i}\right)/\max\,\left(a_{i},b_{i}\right)$$

where *ai* denotes the average dissimilarity of *i* to all members of subgroup A, and *bi* denotes the average dissimilarity of *i* and all members of subgroup B (Meyer et al., 2014). ASW has a range from 0 to 1.

Furthermore, ASW is also calculated with regard to functional experience and boundary-spanning experience (Christopher et al., 2010).

#### Subgroup Balance

The subgroup size is clarified by ASW. Subgroup balance is tested by computing the standard deviation of subgroup size and then multiplying the result by −1 (Carton and Cummings, 2013; Chung et al., 2015).

#### Number of Subgroups

Average silhouette width is used to identify the number of subgroups.

## Method and Results

The fuzzy-set qualitative comparative analysis (fsQCA) is applied so as to examine the extent to which the five antecedent conditions (functional experience diversity, boundary-spanning experience diversity, faultline strength, balance of experience-based subgroups, and number of experience-based subgroups) interact to influence firms’ ability to innovate in BTM&A. FSQCA uses comparative cause, boolean logic and algebra (Judge et al., 2020) to explore the “joint effect” of the interaction processes between multiple factors on a particular phenomenon. There are three main reasons to apply fsQCA. First, while regression analysis explores the “net effect” of a single factor, fsQCA can find group relationships between multiple factors (Rihoux and Ragin, 2009). Second, fsQCA captures the subtle effects of changes in the antecedent conditions at different levels or degrees (Rihoux and Ragin, 2009). Third, fsQCA is flexible when it comes to sampling size (Ragin, 2008).

Based on the purpose of the study, we applied the following three steps for the fsQCA analysis. The first step focused on the calibration of the variable. According to the fsQCA method, the conditions and outcomes should be transformed into fuzzy sets scores, which range from 0 to 1. Using the direct calibration method employed by Ragin (2008), four conditions (functional experience diversity, boundary-spanning experience diversity, faultline strength, balance of experience-based subgroups) and outcomes (BTM&A innovation) are calibrated by defining three anchors. These anchors are the upper quartile (full membership), the median (crossover point of maximum ambiguity regarding membership), and the lower quartile (full non-membership). Meanwhile, the other condition, the number of experience-based subgroups, may lead to null values in the calibrated results, if they have the same upper quartile and median. To prevent this, three calibration anchors are set for the number of knowledge subgroups with full membership, crossover point, and full non-membership as the maximum, mean, and minimum values of the descriptive statistics, respectively. Table 1 shows the calibration and descriptive statistics for each variable.

**TABLE 1:** The calibration and descriptive statistics.

	Calibration			Descriptive statistics			
Condition	Full membership	Crossover point	Full non-membership	Mean	Standard deviation	Minimum	Maximum
Innovation	10	3	0	8.294118	14.74634	0	86
Functional experience diversity	0.61	0.54	0.48	0.534824	0.104899	0.15	0.86
Boundary-spanning experience diversity	0.62	0.57	0.49	0.54911	0.083562	0.29066	0.65778
Faultline strength	0.91	0.83	0.73	0.819529	0.105951	0.55	1
Balance of experience-based subgroup	1	0.73	0.56	0.87265	0.466039	0.28006	2.6833
Number of experience-based subgroup	6	5.42	3	5.423529	0.787796	3	6

The second step provided an analysis of the necessity of the condition variable. The necessity of the condition variable means that the condition always occurs along with outcomes. in our article, a necessity test was carried out in order to identify potential necessities for achieving high innovation in BTM&A. In fsQCA, consistency is an important measure of necessity. More precisely, when consistency exceeds 0.9, the condition is considered necessary for the outcome (Schneider and Wagemann, 2012). Table 2 shows the results of the test for the necessary conditions for high innovation in BTM&A. As can be seen from Table 2, no antecedent condition has a consistency value above the recommended benchmark of 0.90, suggesting that there are no necessary conditions. Thus, high innovation in BTM&A is related to multiple connected factors.

**TABLE 2:** Necessity analysis for high innovation performance.

Conditions	High innovation performance	
	Consistency	Coverage
Functional experience diversity	0.53588	0.531207
∼ Functional experience diversity	0.5625	0.601188
Boundary-spanning experience diversity	0.57662	0.594369
∼ Boundary-spanning experience diversity	0.505556	0.518888
Faultline strength	0.575463	0.585492
∼ Faultline strength	0.509954	0.530332
Balance of experience-based subgroup	0.59676	0.6096
∼ Balance of experience-based subgroup	0.490278	0.507792
Number of experience-based subgroup	0.740741	0.556715
∼ Number of experience-based subgroup	0.375463	0.611614

The third step was the sufficiency analysis of the fsQCA. Unlike the necessity analysis described above, the sufficiency analysis attempts to reveal the sufficiency of the results based on different configurations consisting of multiple conditions. According to a set-theoretic perspective, the analysis of sufficiency explores whether the set of multiple conditions is a subset of the set of outcomes. The truth table algorithm in fsQCA is used to assess sufficiency. The sufficiency analysis uses the five non-necessary antecedent conditions (functional experience diversity, boundary-spanning experience diversity, faultline strength, balance of experience-based subgroups, and number of experience-based subgroups) and the outcome (high innovation in BTM&A). The threshold for consistency is 0.8 (Rihoux and Ragin, 2009), while a score of 1 is used for the frequency threshold due to the small sample size (Greckhamer and Fiss, 2013).

The fsQCA software outputted three complexity assessments: complex (no logical remainders used), parsimonious (all logical remainders used), and intermediate (logical remainders that are consistent with theoretical and practical knowledge used). In contrast with complex and parsimonious solutions, intermediate solutions combine both theories and experience (Schneider and Wagemann, 2012), while also balancing complex and parsimonious solutions (Ragin, 2008). Thus, the intermediate solutions are given here, alongside the parsimonious solutions (Fiss, 2011). Table 3 shows the configurations formed by the five conditions for high innovation in BTM&A.

**TABLE 3:** Configuration for achieving high innovation performance.

Conditions	Configurations			
	C1	C2	C3	C4
Functional experience diversity	🌑	🌑	⊗	⊗
Boundary-spanning experience diversity	🌑	🌑		🌑
Faultline strength	🌑	⊗	●	🌑
Balance of experience-based subgroup	⊗		🌑	
Number of experience-based subgroup	●	⊗	⊗	⊗
Consistency	0.823944	0.806991	0.848987	0.93428
Raw coverage	0.111721	0.126761	0.11005	0.12557
Unique coverage	0.060635	0.075674	0.047028	0.05156
Solution coverage	0.318931			
Solution consistency	0.824182			

*Black circles indicate the presence and crossed-out circles indicate the absence of condition; large circles represent core conditions, and small circles stand for peripheral conditions; blank space indicates a state of ambiguity (the condition may be present or absent).*

Table 3 shows four configurations for achieving high innovation (C1, C2, C3, and C4). The overall solution coverage score is 0.32, which is above the threshold of 0.3 for the coverage of the overall configurations. The level of consistency exceeds the 0.8 threshold for both individual configurations and the overall configurations. The four configurations are sufficient combinations of conditions for Chinese firms to achieve high innovation in BTM&A. More precisely, the first configuration (C1) – “the dominated multiple diversities,” represents TMTs with heterogeneous functional experience and boundary-spanning experience, strong faultlines, imbalanced experience-based subgroups, and more subgroups. In C1, the four conditions (functional experience diversity, boundary-spanning experience diversity, faultline strength, and experience-based subgroup imbalance) are all core conditions. Meanwhile, the number of experience-based subgroups is a peripheral condition. The second configuration (C2) – “the non-aligned multiple diversities,” represents TMTs with heterogeneous functional experience and boundary-spanning experience, weak faultlines, and a few experience-based subgroups. In C2, functional experience diversity, boundary-spanning experience diversity, faultline strength and the number of experience-based subgroups are all core conditions. The third configuration (C3) – “the balanced similarity,” represents TMTs with strong faultlines and balanced experience-based subgroups, but few subgroups and homogeneous functional experience. In C3, highly balanced experience-based subgroups, a few experience-based subgroups, and homogenous functional experience are all core conditions, whereas strong faultlines is a peripheral condition. The fourth configuration (C4) – “the aligned single diversity,” represents TMTs with homogenous functional experience, heterogeneous boundary-spanning experience, strong faultlines, and a few experience-based subgroups. In C4, these four conditions are all core conditions.

## Theoretical Configurational Propositions

Drawing on social categorization and information/decision-making perspectives, this study proposes that diverse TMT attributes and perceptions of subgroup categorizations combine to form configurations for predicting boundary-spanning innovation. The findings reveal that four configurations of factors contribute to high boundary-spanning innovation. It is striking that each of the four configurations includes high or low diversity vis-à-vis boundary-spanning experience. One possible explanation for this is that boundary-spanning innovation is strongly related to either homogenous or heterogeneous TMT boundary-spanning experience. The four configurations are explained in detail below.

For configuration 1 – “the dominated multiple diversities” (heterogeneous functional experience; heterogeneous boundary-spanning experience; strong faultlines; and imbalanced experience-based subgroups), salient subgroup categorization and diversity in both boundary-spanning experience and functional experience do not always harm boundary-spanning innovation. When subgroups are imbalanced, perceptions of diversity in boundary-spanning experience and functional experience are influenced by the extent to which particular subgroups can represent the entire team. In line with the in-group projection model, an existing majority subgroup facilitates boundary-spanning recognition, processing, and integration (Bunderson and Reagans, 2011) and helps boundary-spanning to converge at the team level. There is a common sense between the majority subgroup and small subgroup that small subgroup is generally dominated and tend to “follow” and “obey” to majority subgroup. This reduces unnecessary conflict and competition between subgroups, thus promoting the efficiency of boundary-spanning innovation. At the same time, having a majority subgroup allows knowledge from most team members to be treated fairly due to in-subgroup favoritism. This can further promote boundary-spanning innovation by facilitating wider team knowledge elaboration. When external knowledge adaptation or processing is highly uncertain, members from minority subgroups tend to identify more with the ideas of majority subgroups because the majority subgroup is considered representative of the TMT. At the same time, the majority subgroup encourages cognition and behavior that are simple, clear, unambiguous, prescriptive, focused, and consensual, rather than vague, ambiguous, and unfocused (Reid and Hogg, 2005). Such a method can effectively reduce uncertainty. Some studies highlight that imbalanced subgroups and strong faultlines can discourage minority subgroups from sharing their unique ideas, as they differ from those of the majority (Bowen and Blackmon, 2003). However, this situation can be reversed and alleviated using fixed subgroup boundaries. Under such conditions, members from the majority subgroup tend to respect and develop the ideas of the minority subgroup in order to keep the team unified, because members of the majority subgroup do not need to worry about the members of the minority subgroup challenging their prototypical position. This is in line with Gordijn et al. (2002), who note that the superordinate category tends to process minority knowledge as issue-related and divergent thinking when the minority subgroup is consistent rather than inconsistent.

For configuration 2 – “the non-aligned multiple diversities” (heterogeneous functional experience; heterogeneous boundary-spanning experience; weak faultlines; and a few experience-based subgroups), when unidimensional experience diversity is transformed into multi-dimensional experience diversity, weak faultline benefit, rather than harm, boundary-spanning innovation. One possible explanation for this is that boundary-spanning innovation may not always give rise to high uncertainty or risk. When the uncertainty or risk is low, any reduction of uncertainty/risk by subgroup categorization no longer has psychological importance (Reid and Hogg, 2005). Furthermore, attention can then shift from between-subgroup comparison and in-subgroup support to individuals’ differences in functional experience and boundary-spanning experience. In addition, the less salient the boundary-spanning experience and functional experience, the lower the likelihood that members will recognize out-subgroup hostility (Lau and Murnighan, 2005). As a result, harmony vis-à-vis within-team knowledge elaboration is enhanced, which promotes boundary-spanning innovation. Although a weak faultline limits subgroup support to members seeking to freely express their own ideas, it also prevents informational segmentation (Lau and Murnighan, 1998). For functional diversity, there is an especially robust literature that demonstrates that salient functional diversity helps information processing, mainly based on personal purpose rather than the team-oriented purpose (e.g., Harvey, 2015). Limiting the number of subgroups increases members’ attention and promotes cognitive convergence on solving key questions facing boundary-spanning innovation.

For configuration 3 – “the balanced similarity” (homogenous functional experience; highly balanced experience-based subgroups; and few experience-based subgroups), homogenous functional experience facilitates cognitive convergence on creative boundary-spanning knowledge integration, but fails to provide diversity in functional knowledge for boundary-spanning knowledge “collision.” Therefore, TMTs with homogenous functional experience need to rely on subgroup support in order to widen opportunities for debates and dialectical exchanges for the purpose of integrating homogeneous knowledge. In line with arguments for in-group projection and attention concentration, the presence of a few evenly sized subgroups enhances individuals’ willingness to present and challenge ideas relating to boundary-spanning knowledge management based on similar functional experiences. This is because it is easier for team members to be confident in the prototypical nature of their own knowledge and to focus on details and dissimilarities vis-à-vis others’ homogeneous knowledge (Xie et al., 2015). When debates and dialectical exchanges involving boundary-spanning knowledge from functional experience-based subgroups surface at the team level, they can facilitate knowledge exchange and processing through specialization, increased attention to dissimilar ideas, and an enhanced ability to locate knowledge in the team, and the in-depth analysis of boundary-spanning knowledge. All of this in turn promotes boundary-spanning innovation. More notably, subgroups must remain weakly or moderately salient (the peripheral condition for faultline strength). One possible explanation for this is that, in some cases, integration at the P-M&A stage is highly successful, which reduces uncertainty and risk in innovation. Therefore, individuals do not need to rely on subgroup categorization for the purpose of reducing uncertainty and risk. Moreover, strong subgroup salience can lead to negative stereotypes being developed with regard to the out-subgroup, thereby increasing conflict between subgroups (Lau and Murnighan, 1998). For subgroup categorization in homogenous teams, members find it easier to perceive knowledge overlap or redundancy between subgroups compared to heterogeneous teams. Positive subgroup distinctions are hampered when within-team similarities can be easily identified, but within-team differences are seldom recognized (Hornsey and Hogg, 1999).

For configuration 4 – “the aligned single diversity” (homogenous functional experience; heterogeneous boundary-spanning experience; strong faultline; few experience-based subgroups), the creation of experience-based faultlines depends predominantly on heterogeneous boundary-spanning experience rather than homogeneous functional experience. More precisely, in-subgroup support and out-subgroup comparison are dominated by heterogeneous boundary-spanning experiences. Different from most circumstances of strong faultline, the stronger the faultlines in this situation, the stronger the subgroup support, but the less likely the negative stereotypes of the out-subgroup. The reason for this is that team members tend to show high tolerance and understanding for between-subgroup ideas regarding how to evaluate boundary-spanning knowledge. In boundary-spanning M&A, externally acquired boundary-spanning knowledge entails high risks and uncertainty as a result of the “not invented here” syndrome (Bresman and Zellmer-Bruhn, 2013). When boundary-spanning experience-dominated faultlines exist with less dividing lines (fewer subgroups), in-subgroup support and out-subgroup boundary-spanning variance understanding may help TMTs to cope with threats and concentrate on subgroup-based processing of boundary-spanning knowledge, further enabling members to manage externally acquired knowledge (Xie et al., 2015). This view is in line with the sharing model of boundary-spanning, which suggests that establishing dynamic alignment along with common means and interests between different domains of employees can facilitate boundary-spanning knowledge sharing and elaboration (Hsiao et al., 2012). As a result, the formation of faultlines based on boundary-spanning diversity leads to strong TMT specialization, which in turn leads to an increase in boundary-spanning innovative activities.

## Discussion

### Theoretical Implications

This study examines the antecedent configuration of boundary-spanning innovation – boundary-spanning diversity, functional diversity, faultline strength, the number of subgroups, and subgroup balance, thereby contributing to the diversity and configuration literature in the following ways. First, although boundary-spanning innovation has become a major target in the P-M&A stage, few studies have explored the antecedents of boundary-spanning innovation in the diversity context. Despite the few pioneering studies on related behaviors, such as boundary-spanning activities (e.g., Zhang et al., 2017), this line of research is still in its infancy and limited in theoretical development. This study contributes to the literature by developing an integrative framework vis-à-vis the antecedents of boundary-spanning innovation and confirms long-standing beliefs with regard to the net causal relationship between unidimensional diversity and innovation, in which experience-related diversity exerts an inconsistent influence on the team or organizational innovation (Van Knippenberg et al., 2004).

Second, rather than proposing a specific antecedent for boundary-spanning innovation, the fsQCA findings provide evidence to support the presence of causal complexity, as four paths lead to relevant outcomes in boundary-spanning innovation. All four paths can be regarded as complementary and equally important because each provides a unique but sufficient path to innovation. These findings differ from linear regression models on innovation and its antecedents, which only suggest a significant association between variables. The configurational approach can improve our understanding of the connections between antecedents, such as their complementarity or suppression (Leischnig and Kasper-Brauer, 2015). Although the findings provide evidence that four configurations can explain high boundary-spanning innovation, the linear regression models are also useful for clarifying the degree to which organizations use different antecedents of innovation. Therefore, a combination of both analytical approaches merits needs to be further explored.

Third, this study extends the literature on the integration of social categorization and information/decision-making perspectives by integrating these two perspectives within the configurational framework, thereby shedding light on the complex connection between unidimensional diversity and multidimensional diversity. The four configurations represent an important qualification of both theoretical perspectives, as they show that subgroup categorizations do not necessarily hamper knowledge-related innovative activities among work experience differences in all cases. More precisely, differences in functional experience and boundary-spanning experience may provide a broader range of knowledge or information with regard to the team or organizational innovation when both types of experience exist without subgroup salience and numerical increase. Meanwhile, the situation is different when faultline strength, the number of subgroups, and the level of diversity are changed. With homogeneous functional experience, faultline strength and subgroup balance separately motivate subgroup-based informational elaboration. Even when multiple working experience diversities along with subgroup exist, the majority subgroup takes on a prototypical role in promoting a team or organizational innovation. Such a finding is in line with assumptions from the in-group projection model. And this in-group projection model assumes that the larger the subgroup majority, the more likely they will form the prototype (Hogg et al., 2012). Overall, this study notes that boundary-spanning innovation occurs based on multiple theoretical perspectives. Future studies should make use of and expand on this configurational framework. At the same time, this study addresses calls from diversity scholars to pay more attention to the causal complexity within the categorization-elaboration literature (e.g., Hoever et al., 2012) and to shed light on the significance of subgroup categorization when managing team diversity (Mo et al., 2019).

### Practical Implications

This study also has practical implications for organizations, which are centered around configurational findings. First, given that high boundary-spanning innovation is dependent on the attributes of the boundary-spanning experience and functional experience, it is important to consider which approach should be adopted when composing TMTs vis-à-vis configurations of homogeneous or heterogeneous factors. More precisely, practitioners need to identify the core antecedents and notice those antecedents that are absent. TMTs specialized in core diversity-related factors will then foster innovation, whereas TMTs specialized in absent diversity-related factors will have no positive effect on innovation. TMTs can be designed in such a manner that produces salient and imbalanced subgroups that have diverse boundary-spanning experience and functional experiences. However, it is unrealistic to expect TMTs to be designed in such a way that avoids the formation of subgroups because this will prevent organizations from selecting highly talented team members with diverse work experience (Georgakakis et al., 2017).

Second, for boundary-spanning innovation, TMT boundary-spanning diversity and the number of subgroups play an important complementary role. For instance, in most configurations, when leaders focus on facilitating innovation, the preferred TMT should include boundary-spanning diversity. Doing so gives the team a large pool of knowledge to deal with external knowledge, and focuses their attention on key issues in boundary-spanning innovation.

Third, the configuration solutions in this study suggest different HR strategies for establishing a well-functioning TMT. All of which are equally effective for achieving boundary-spanning innovation. Therefore, the solutions not only highlight the futility of trying to develop a “perfect formula” for structuring TMTs but also offer practical guidelines for re-allocating team members.

### Limitations and Future Research

Despite its impressive contribution, there are several limitations in this study. First, it is appropriate to apply fsQCA in the study of complex causal relationships, but this method is more appropriate in certain contexts. For instance, as fsQCA is related to fully interactive models which consider all possible configurations, its data matrices increase exponentially as more causal situations are added. Accordingly, the number of cases available limits the number of causal conditions that can be analyzed simultaneously, and any studies should ensure sufficient degrees of freedom to avoid over-determined results (Fiss, 2011). Second, this study only explores the antecedents of high innovation in BTM&A at the micro level of TMTs. In addition, equity characteristics and R&D investment at the firm level, as well as competitive pressures and the market environment at the industry level, are also important factors that influence corporate innovation. Future research should consider these additional factors further. Third, this study uses applied patents instead of sales revenues from new products. This is because the organizations selected do not offer such granular data. Hence, it is difficult to obtain data on new product sales. Future studies should try to examine sales revenue for new products to see if the same findings are subsequently produced.

## Data Availability Statement

The original contributions presented in this study are included in the article/supplementary material, further inquiries can be directed to the corresponding author.

## Author Contributions

MQ, XL, and WW were all instrumental in the development of the research project, made significant contributions to the overall writing, and theoretical development of the manuscript. XL carried out the data collection and ran the statistical analyses. All authors contributed to the article and approved the submitted version.

## Conflict of Interest

The authors declare that the research was conducted in the absence of any commercial or financial relationships that could be construed as a potential conflict of interest.

## Publisher’s Note

All claims expressed in this article are solely those of the authors and do not necessarily represent those of their affiliated organizations, or those of the publisher, the editors and the reviewers. Any product that may be evaluated in this article, or claim that may be made by its manufacturer, is not guaranteed or endorsed by the publisher.
